# Systemic Review: Ozone: A Potential New Chemotherapy

**DOI:** 10.3390/ijms222111796

**Published:** 2021-10-30

**Authors:** Jose Baeza-Noci, Rosa Pinto-Bonilla

**Affiliations:** Department of Human Anatomy & Embryology, School of Medicine, University of Valencia, 46010 Valencia, Spain; rosa.pinto@uv.es

**Keywords:** ozone therapy, oncology, cancer

## Abstract

In the last sixty years, publications in reputed journals have shown the preclinical positive effect of ozone gas in cancer cells. However, the translation of these results into clinical practice is far away from success. A comprehensive approach is necessary for this, and oncologists and researchers need guidance from medical specialists with in-depth knowledge of ozone in medicine. In this article, we review the evidence around this question and suggest different potential research lines to those interested in this exciting field.

## 1. Introduction

According to 2020 WHO report [1] on global mortality, cancer is the fifth leading cause of death, and it has been increasing in the past 20 years, whereas cardiovascular diseases and infections are decreasing year after year. If we check the leading causes of death by income group (gross product) and their evolution in the past 20 years, we will realize that this trend is common to all income groups, and in the high-income countries, cancer is the third cause of death after heart attack and dementia. In the next years, we will see cancer as the second cause of death. This will be because of two main reasons: the increase in life expectancy—cancer is directly related to aging—and the decrease in risk factors for heart infarction and better treatments for it.

The mortality rate in cancer is globally estimated by the WHO [2] to be approximately 50%, although this rate seems to depend on the country’s gross product; wealthy countries have a mortality rate that ranges from 20% to 48%, whereas poor countries have a rate over 50%. We can understand how early detection, top health technology and new treatments can influence this fact. Mortality trends, when compared with incidence trends, can also provide evidence of the impact of improved treatments, including screening and early diagnosis. If death rates drop faster than incidence, this may reflect the availability of better tools for cancer diagnosis and treatment.

In the United States [3], the cancer death rate has declined since the 1990s. Although death rates for many cancer types have also decreased, rates for a few cancers have not changed or even increased. This fact shows the necessity of reducing risk factors that are rising (obesity, sedentarism) and investigate new treatments.

Although the classic approach of surgical removal followed by radiotherapy (RT), chemotherapy (CT), or both is still valid for many cancer types, new treatments, such as immunotherapy, show promising results.

RT and some CT drugs [4] induce cancer cell death by increasing Reactive Oxygen Species (ROS) and free radicals inside tumor cells. Due to the well-known but still mysterious Warburg effect (aerobic glycolysis or aerobic fermentation of glucose) and its consequences [5], the cancer cell has a very delicate adaptation to an increased ROS production, and any external induction of more ROS could break this balance, triggering cell apoptosis. Cancer cell-increased ROS production is due to a greater intake of glucose and the Warburg effect. 

On the other hand, the adaptation is achieved by increasing the synthesis of antioxidants enzymes and enhancing some metabolic pathways (e.g., the pentose phosphate pathway coming from glycolysis generates NADPH) that balance this excess ROS. This consequence of the Warburg effect may be directly involved in oncogene-induced senescence (OIS). OIS has a tumor-suppressive cellular function, and a recent study has reported that increased glucose oxidation through pyruvate dehydrogenase (PDH) can regulate OIS. 

ROS have been reported to be tumorigenic, as they increase cell proliferation, survival, and cellular migration. They induce chromosome damages that initiate tumorigenicity and tumor progression by inactivating phosphatase and tensin homolog (PTEN) and tyrosine phosphatases. On the other side, ROS can also produce cell membrane damage and other deleterious effects that lead to cell death [6].

ROS inhibition has proven to block the anti-cancer effect of cisplatin and rapamycin [7], and a selective ROS induction with photodynamic therapy has also shown a targeted tumoricidal result [8].

**Figure 1:**
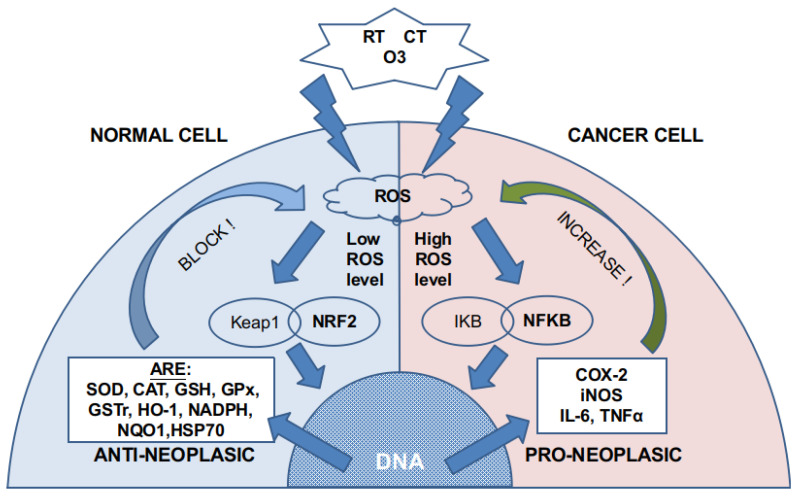
ROS generation by RT, CT, and ozone. According to previous cytoplasmic and mitochondrial ROS levels, different pathways are activated. Normal cells have a low ROS level, whereas cancer cells have high or very high ROS levels. The NRF2 pathway is almost exhausted in cancer cells, so more ROS activate the NFKB pathway.

Ozone can directly damage the cell membrane [9] by oxidizing the fatty acids (lipid peroxidation) that compose it. This reaction (Criegee’s reaction) induces [10] the generation of hydroperoxides, mainly hydrogen peroxide (H_2_O_2_), aldehydes such as malonyl dialdehyde (MDA), alkenes such as 4-hydroxynonenal (4-HNE), and lipoperoxides (mainly 9 alfa-hydroxy-hydroperoxide) that are partially tampered by antioxidant enzymes placed in the inner layer of the cell membrane. Molecules that are not reduced react with cytoplasm molecules such as NADPH. If there is not enough NADPH, as it may happen in cancer cells, they induce signaling and damage to the cell by producing acute oxidative stress. In non-cancer cells, these molecules produce an activation in nuclear factor NFR2 that increases the synthesis of antioxidant molecules and induces a modulation over nuclear factor NFKβ. As cancer cells have an overloaded antioxidant system because of the increased ROS level, they have a small capability of increasing, even more, their production of antioxidants. This is the reason why non-cancer cells can handle safely ozone doses that are toxic for the cancer cell (Figure 1). Moreover, there is a concomitant increase in membrane permeability that also creates an alteration in the ion concentration of the cytoplasm fluid inducing cell apoptosis. 

High-impact journals have published during the past sixty years interesting papers supporting the in vivo and in vitro role of ozone (O_3_) in inducing direct cancer cell damage in a harmless way for non-cancer cells. However, few clinical articles have been published, and so, there is small evidence-based support for its clinical use in cancer patients.

We address this paper to compile and analyze this literature and suggest the possible use of ozone as a chemotherapeutic agent, both locally or in a systemic way, alone or associated with CT and/or RT protocols. Potential new investigational lines can also be suggested.

## 2. Material and Methods

**Figure 2:**
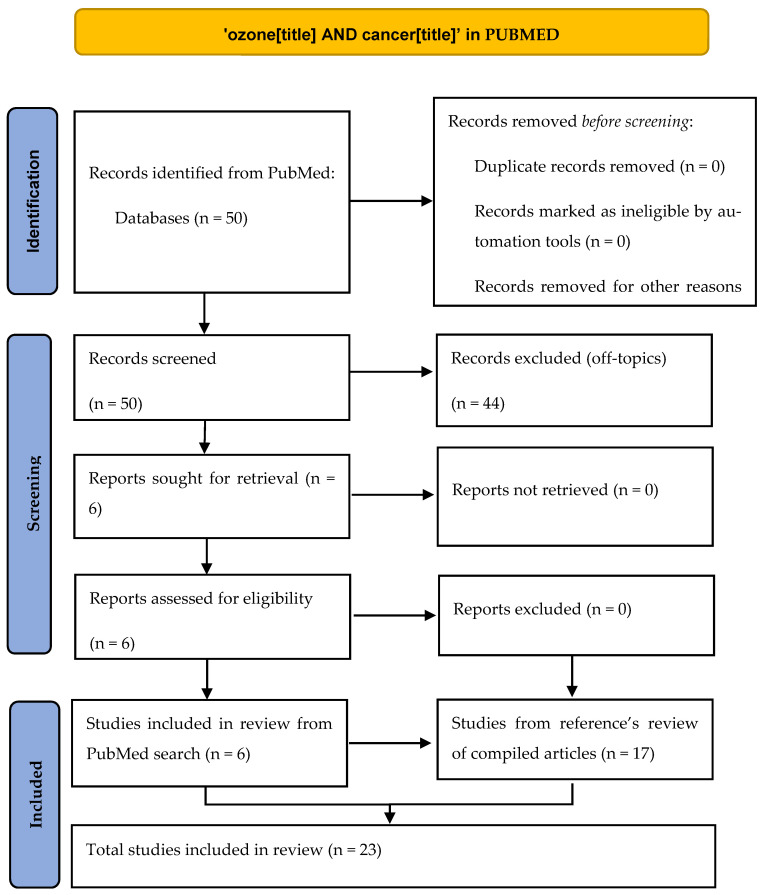
PRISMA scheme of our review.

Our target has been to find articles in which ozone has been used to directly kill cancer cells according to the mechanism explained in the Introduction section of intracellular ROS increase, whether in vivo, in vitro, or in human patients. We found this approach interesting after reading two reviews [4,11] about the potential role of ozone therapy in oncology as it can be extremely safe for non-tumor cells. To fulfill our aim, we have done a systematic review following PRISMA guidelines [12] by searching in the PubMed database the terms ‘ozone AND cancer’ in the ‘title’ field, finding 50 references. After a critical reading of title and abstract, only 6 references fulfilled our scope (Table 1 and Figure 2), and we read all of them (5 in vitro studies, 1 in vivo study). In order to widen our literature review, we did a detailed reading of the references in the papers found in the first search. From this reference’s scan in these papers and also in the two review articles mentioned above, we found (Table 2) 17 more articles (6 in vitro studies, 8 in vivo studies, 3 clinical studies). As we found very different kinds of articles (preclinical controlled, preclinical non-controlled, case series), a PRISMA checklist could not be fully accomplished, so we do not consider our paper as a strict systematic review but rather an evidence review. All 23 works are reflected in this paper and have been explained in detail both in the tables and in the main text of this article.

## 3. Results

### 3.1. In Vitro Studies

In 1958, Fetner [13] published in *Nature* the role of ozone gas in inducing chromosome breakages similar to those effects observed with X-ray irradiation and also the additive effect of X-ray and ozone gas in cell culture. Four years later, the same author [14] confirmed his first observations in a new article using a HeLa cell culture (cervical uterine adenocarcinoma). 

In 1980, Sweet et al. [15] described in *Science* how ozone selectively inhibited, in cell cultures, the growth of three different cancer cell lines (breast, lung, and uterus) without affecting nontumor cells; this effect is related to the ozone dosage and possibly explained because of a defective enzymatic pathway in cancer cells related to the reduced glutathione (GSH) respiratory linked system. 

Seven years later, a work from Karlic et al. [16] described a cytotoxic effect of ozone on three ovarian carcinoma cell lines but showed no effect in one endometrial carcinoma cell line, using the same ozone dosage. Irradiation (Ra226, Ir192, or Co60) was used alone and in combination with ozone, finding a radiosensitizing effect of ozone. Control normal cells (skin fibroblasts) had no changes, even with ozone and irradiation with Ra226, but they were damaged by Ir192 or Co60.

In 1990, ozone was described by Zanker and Kroczek [17] as having a similar effect to chemotherapeutic drugs by increasing ROS inside culture cells. They also noticed a synergistic or additive effect on 5-fluorouracil (5-FU) in breast cancer and colon cancer cell lines; even more, ozone overcame chemoresistance to 5-FU in cell lines previously resistant. Ozone and 5-FU did not affect glioma cells neither alone nor together. 

That year, Washuttl J. et al. [18] demonstrated that ozone induced damage in the respiratory pathway used by cancer cells (ovarian carcinoma) that induced apoptosis; this damage is similar to the one produced by Doxorubicin or Ifosfamide and does not happen in healthy ovarian tissue.

In 2007, Cannizzaro et al. [19] founded a direct effect of ozone in two neuroblastoma cell cultures (SK-N-SH and SK-N-DZ), in which ozone reduced the cell growth or induced cell apoptosis. In SK-N-SH cells, there was a potentiated effect by combining ozone and cisplatin or etoposide but not with gemcitabine. In SK-N-DZ, only ozone could inhibit the growth of cancer cells.

Simoneti et al. [20] described in 2017 a direct cytotoxic effect of ozone in a human colon cancer cell culture and an increased effect of 5-fluorouracil or cisplatin by combining them with ozone.

One year later, Mokhtari et al. [21] checking the use of Cold Plasma-Activated Media (PAM) found that the underlying mechanism of cancer cell cytotoxicity was the generation of ozone because of the PAM. Six cancer cell lines were tested, and the cytotoxic effect was different. Increasing the PAM time application induced more ozone generation and so, more cytotoxicity. The colon cancer line was the less affected one, and breast cancer was the most damaged line. The authors suggest that the ROS generated by ozone trigger signaling pathways involving c-Jun NH2-terminal kinase (JNK) and p38 kinase and promote mitochondrial perturbation, leading to apoptosis. 

Other authors [22] have verified that low ozone concentration did not induce any changes in HeLa cell cultures, suggesting that ozone dose may be important to achieve an antitumor effect.

Lately, Li et al. [23] verified that ozone gas on hepatocarcinoma (bel7402 and SMMC7721 cancer cell lines) cell cultures restrains the proliferation and migration potential thanks to the increase of ROS and the NFKβ suppression.

To summarize this section, ozone gas in normal and cancer cell cultures has variable effects: a direct cytotoxic effect on some types of tumors (not all) while not damaging normal cells when used at the same dosage; there is an additive or synergistic effect with RT and some CT drugs, which is probably related to the intracellular increased production of ROS and other free radicals. This increase in intracellular ROS is badly handled by tumor cells but causes no effect in normal cells due to their intact capability of increasing antioxidants.

### 3.2. In Vivo Studies

All the experiments developed on animal models [24,25,26,27,28,29,30,31,32] have not used a direct approach in the sense of a direct gas administration into the tumor, as the in vitro model. Ozone has been given through a systemic way (rectal ozone insufflation and intraperitoneal mainly) and does not reach directly the tumor. Instead, secondary messengers, such as 4-hydroxynonenal (4-HNE), H_2_O_2_, and lipoperoxides (mainly 9 alpha-hydroxy-hydroperoxide) generated from the Criegee’s reaction between ozone and polyunsaturated fatty acids placed in albumina are distributed in the whole body [8]. These molecules induce a modulation in nuclear factor NRF2, regulating the Antioxidant Response Elements (AREs) [33], modulating the nuclear factor NFKβ that plays a basic role in inflammation and immune response, and increasing inside erythrocytes 2,3-diphosphoglycerate (2,3-DPG) and ATP, originating an enhanced gas exchange both in lungs and in peripheric tissues and an improvement in the blood flow.

All these mechanisms improve immunity [23,24,33] and increase radiosensibility [25,26,27,28,29,30] in some tumors, but they are not the target of this paper.

Only Kuroda’s team [34] has published a study in an animal model (a mouse with rectal cancer tumor-bearing) showing necrosis and the inhibited proliferation effect of an intratumor injection of 1 mL of ozonated water (ozone dissolved in bi-distilled water at concentrations of 20.8, 41.6, 104, and 208 mM in four groups of mice), compared with 1 mL of bi-distilled water and no treatment. They injected 1 mL of ozonated water at a 208 mM concentration subcutaneously, intraperitoneally, and intramuscularly (0.1 mL) for three days. Ozonated water caused no change in healthy tissues. Higher ozone dosage induced more tumoral necrosis without damaging the normal tissue. These results support the in vitro experiences, but more investigation is needed in this area.

### 3.3. Clinical Works

The only paper we have found in our suggested line is from Megele et al. [35] that showed increased survival (30.5 months compared with the standard value of 11.9 months) in a series of four patients with recurrent glioblastoma that were treated with re-resection of the tumor (after relapsing) and intratumoral ozone administered monthly through a catheter (5 mL of oxygen–ozone gas at a concentration of 40 μg of ozone per mL of oxygen—a total dose of 200 μg each time), together with the standard protocol of chemotherapy. The patients received a median of 27 (range: 3–44) oxygen–ozone applications. Another patient was treated with ozone just after the first surgery (together, the RT and CT protocols used in all the patients were included in this case series); he is still alive and without recurrence after 53 months. Two side effects were reported; one catheter was removed temporarily because of an infection and another one, in a different patient, was removed because of a hemorrhage.

Other clinical papers, mainly case reports or short case-control series, have suggested a collaborative effect of ozone with RT [36] or CT [24,37]. The oxygenating effect of ozone [10] can increase the radiosensitivity of some tumors, improving the survival rate. The modulation on the immune system induced by ozone [10] may play a role in enhancing the anti-cancer effect of other drugs.

## 4. Discussion

RT and many CT drugs have an anti-tumor effect mediated by the production of ROS and free radicals in tumor cells. ROS inhibition decreases CT activity [8], and a selective ROS induction increases tumoricidal result [9].

These ROS induce DNA alterations and other signaling molecules that produce cell apoptosis. In vitro studies have compared the anti-tumoricidal ability of ozone-compared to X-ray [13,14] but also to well-known RT procedures, such as irradiation with Ra226, Ir192, or Co60 [16]. Ozone, at the right dosage, seems to induce the same cellular effect without damaging the normal tissue cells [15] because the antioxidant system in these cells can usually handle this injury, whereas cancer cells have an almost exhausted antioxidant capacity. It is interesting to note that ozone has a radio-sensitizing effect when used with some RT protocols in vitro, turning radiosensible tumor cells that were radioresistant [16]. 

This radio-mimetic effect has been observed also when comparing ozone with some CT drugs, such as 5-FU [17,20], doxorubicin, or ifosfamide [16]. Ozone induces a similar cancer cell damage to that produced by these drugs by increasing ROS in tumor cells [23]. Moreover, there is a potentiated or synergistic effect when using these CT drugs and ozone together [17,19], the same as what we can observe with the above commented RT protocols. This synergistic effect seems to be different according to the diverse CT drugs used [19] (cisplatin, etoposide, or gemcitabine) and may be different on diverse cancer cell lines.

Recent papers, such as the ones from Mokhtari [21] or Li [23], give us more detailed information about the biochemical pathways used by ROS to induce cell apoptosis in cancer cells.

According to this review, not all tumors are equally susceptible to these potential treatments. We have seen that HeLa cell line is damaged [14] or preserved [22] according to the ozone dose.

These RT-like and CT-like effects have been checked in vitro, but in vivo models are very scarce [34]. Kuroda has checked the efficacy and safety of ozonated water in mice at different ozone concentrations without side effects for healthy tissue at the same dose. The only clinical experience from Megele [35] is very interesting but limited. Increasing the survival rate almost three times is an outstanding result but reduced to five patients.

Systemic ozone has a different mechanism of action [24,25,26,27,28,29,30,31,32], as the usual doses have not proved to induce tumoral cell apoptosis. As this helpful way used in some clinical studies has no direct relation with the RT-like or CT-like ozone effect, we will not deepen our study on it. However, thanks to the great antioxidant capability of the normal cells, higher doses of ozone could have a similar effect to topic ozone gas administration to the tumor, causing no harm to rest of the organism. However, no study has been published in this sense.

## 5. Conclusions

The use of ozone in cancer needs still a lot of preclinical investigation not only by testing more cancer cell lines but also testing different ozone dosages, as we presently know that all cancer cell lines are not equally affected by ozone.

In vivo studies have been mainly devoted to systemic ozone with low doses. It is mandatory to develop more animal studies following Kuroda’s investigational line, or with intratumoral ozone gas injection.

Randomized control trials should be the third step, after deeper preclinical studies have been done. To develop clinical studies, different interventional approaches can be used theoretically to dispense ozone into the tumor: specific arterial embolization, intratumoral injection, and catheterization. As a single application of ozone is not likely to affect all tumor cells, several applications of this gas would be needed to progressively affect all the tumor.

To use ozone as a CT agent in a systemic way, further studies testing the tumor cell reaction to ozone in vivo must be done. Work is warranted in this regard.

## Figures and Tables

**Table 1:** Papers retrieved from direct search in PubMed. See Figure 2 for details.

Author (Year)	Study Type	Disease	Route of O_3_ Application	Unit of Analysis	Sample Size	Findings
Sweet F. et al. (1980)	Basic research In vitro	Control: - Human lung diploid fibroblasts. Diseases: - Lung alveolar adenocarcinoma - Breast adenocarcinoma - Uterine carcinosarcoma - Endometrial carcinoma	Topical gas	Cell cultures	5 groups	Control cells suffered no changes while all cancer cells had apoptosis and growth inhibition. At higher doses, control cells had growth inhibition, and cancer cells showed greater damage.
Simonetti V. et al. (2017)	Basic research In vitro	HT29 human cancer colon	Topical gas +/− CT (cisplatin or 5 -fluorouracil (5-FU))	Cell cultures	14 groups: - O_3_ (4 subgroups) - Cisplatin - O_3_ + Cisplatin (4 subgroups) - 5-FU - O_3_ + 5-FU (4 subgroups)	O_3_ damaged cancer cells and has a synergistic effect with both CT drugs.
Mokhtari H. et al. (2018)	Basic research In vitro	Control: - Skin fibroblasts - Mammalian gland cells Diseases: - Breast cancer (SKBR3, MCF7) - Pancreatic cancer (ASPC-1) - Lung adenocarcinoma (A-549) - Osteosarcoma (G-292) - Colon carcinoma (SW742)	Cold atmospheric plasm activated media (PAM)	Cell cultures	8 groups: - 2 controls - 6 diseases	All cancer cell lines were affected by the exposure to PAM, directly related to the exposure time. O_3_ production by PAM was the reason. Colon carcinoma was the most resistant line and breast cancer and the most affected one.
Costanzo M. et al. (2020)	Basic research In vitro	HeLa cell line	Topical gas	Cell cultures	2 groups (O_3_ different doses)	Cancer cells were not affected at these doses.
Li J. et al. (2021)	Basic research In vitro	Hepatocarcinoma (bel7402 and SMMC7721 cancer cell lines)	Topical gas	Cell cultures	2 groups	Ozone restrains the proliferation and migration potential of liver cancer cells via ROS accumulation and PI3K/AKT/NF-κB suppression.
Dogan R. et al. (2018)	Basic research In vivo	Tongue cancer rat model (4NQO)	Rectal insufflation RT	Rats	36: - Cancer - Cancer + RT, - Cancer + O_3_ + RT. - Cancer + O_3_ - Control	O_3_ increases 3 times the survival time compared with RT. RT plus O_3_ prolonged survival time 11 times more than RT alone.

**Table 2:** Papers retrieved from references’ scan of articles in Table 1 plus articles [4,11].

Author (Year)	Study Type	Disease	Route of O_3_ Application	Unit of Analysis	Sample Size	Findings
Fetner R. (1958)	Basic research In vitro	No disease	Topical gas +/− X-ray	Vicia faba seeds	4 groups: - O_2_ - O_3_ - X-ray - O_3_ + X-ray	Compared with O_2_, both O_3_ and X-ray induced chromosome breakages; more together or with higher doses
Fetner R. (1962)	Basic research In vitro	KB cell line (HeLa)	Topical gas +/− X-ray	Cell culture	6 groups: - O_2_ - O_3_ - X-ray - O_3_ + X-ray (3 subgroups)	Compared with O_2_, both O_3_ and X-ray induced chromosome breakages; more together or with higher doses
Grundner HG. et al. (1976)-III	Basic research In vitro	Peritoneal carcinomatosis (Erlich ascites carcinoma)	Topical gas +/− RT	Cell culture	3 groups: - O_3_ - RT - O_3_ + RT	O_3_ inhibits tumor growth and is more effective if associated with RT.
Karlic H. et al. (1987)	Basic research In vitro	Control: - Skin fibroblasts Diseases: - Ovarian carcinoma (OC) - 2 different lines of ovarian adenocarcinoma (OC1, OC2) - Endometrial carcinoma (EC)	Topical gas +/− RT (Ra226, Ir192, or Co60)	Cell cultures	29 groups: - Control - Each disease: O_3_, Ra226, Ir192, Co60, O_3_ + Ra226, O_3_ + Ir192 O_3_ + Co60 (7 subgroups)	Control cells were not damaged by O_3_, Ra226, or both. Ir192 or Co60 alone damaged control cells. Endometrial carcinoma was resistant to O_3_ or Ra226, but affected by the combination. The rest of cancer cell lines were damaged by any RT or O_3_ +/− RT.
Zanker KS. et al. (1990)	Basic research In vitro	- Breast cancer - Colorectal adenocarcinoma - Glioma - Colorectal adenocarcinoma resistant to 5-FU	Topical gas +/− 5-FU	Cell cultures	12 groups: - O_3_, 5-FU and both in each line.	Only glioma cells were not damaged by O_3_, 5-FU or both. In the other cancer cell cultures, O_3_ and 5-FU had a synergistic or additive effect.
Washuttl J. et al. (1990)	Basic research In vitro	Control: - Ovarian healthy tissue (OHT) Disease: - Ovarian carcinomatosis (OC)	Topic gas, CT (Doxorubicin or Ifosfamide)	Cell cultures	6 groups: - Control (OHT/OC) - O_3_ (OHT/OC) - Doxorubicin (OC) - Ifosfamide (OC)	O_3_ does not harm OHT while at the same doses, it induces damage in OC, similar to the produced by the CT. There is damage in the respiratory pathway of OC caused by O_3_ that does not happen in OHT.
Cannizzaro A. et al. (2007)	Basic research In vitro	SK-N-SH and SK-N-DK neuroblastoma	Topical gas +/− CT (Cisplatin, Etoposide, or Gemcitabine)	Cell cultures	8 groups: - Control - O_3_ - CT (3 subgroups) - O_3_ + CT (3 subgroups)	SK-N-SH cells were affected by O_3_, CT and both (synergistic effect). SK-N-DK cells were only affected by O_3_.
Menendez S. et al. (2008)	Basic research In vivo RCT phase III Single blind	Erlich ascites carcinoma + Sarcoma 37 Lewis lung carcinoma Prostatic adenocarcinoma (intracapsular)	Rectal insufflation Intraperitoneal insufflation before the tumor implantation RT (Co60) +/− Rectal insufflation	Mice Mice Patients	50: - Control - 4 different O_3_ doses. 50: - Control - 4 different O_3_ doses. 70: 35 / 35	Significant decrease in the number of metastases in the O_3_ group, which was directly related to the O_3_ dose. Significant decrease in tumor growth in pre-treated O_3_ groups, which was inversely related to the O_3_ dose. Significant decrease in side effects (radio- dermatitis, cystitis, proctitis) and PSA in O_3_ group.
Schulz S. et al. (2008)	Basic research In vivo	Head and neck squamous cell carcinoma	Intraperitoneal insufflation	Rabbits	59	Tumor regression that could be blocked by immunosuppressors.
Rossmann A. et al. (2014)	Basic research In vivo	Head and neck papillomavirus- related cancer	Intraperitoneal insufflation	Rabbits	20	Tumor eradication due to enhanced immunity (increase in CD3+ T-cells).
Hernuss P. et al. (1974)	Basic research In vivo	Walker carcinosarcoma	RT +/− Intraperitoneal insufflation	Rats	Not reported. 2 groups: - RT - RT + O_3_	RT combined with O_3_ produced better outcomes than only RT. Tumor remission was 39% in the RT + O_3_ group versus 0% in RT without O_3_ group.
Grundner HG. et al. (1976)-I	Basic research In vivo	Peritoneal carcinomatosis (Erlich ascites carcinoma)	RT +/− intravenous	Mice	Not reported. 2 groups: - RT - RT + O_3_	O_3_ does not add any inhibitory effect to RT.
Grundner HG. et al. (1976)-II	Basic research In vivo	Peritoneal carcinomatosis (Erlich ascites carcinoma)	RT +/− Intraperitoneal insufflation	Mice	Not reported. 2 groups: - RT - RT + O_3_	O_3_ does not add any inhibitory effect to RT.
Kiziltan HS. et al. (2015)	Basic research In vivo	Peritoneal carcinomatosis (Erlich ascites carcinoma)	Intraperitoneal insufflation +/− RT	Mice	60 3 groups: - RT - O_3_ - RT + O_3_	O_3_ and RT increased the survival rates either separately or concurrently.
Kuroda K. et al. (2015)	Basic research In vivo	Rectal adenocarcinoma	Intratumoral injection of ozonated water	Mice	Not reported. 4 groups: - ? healthy - 5 control - 5 water - 6 O_3_ water	No changes in normal tissues of healthy mice with O_3_ water. Normal growth of cancer cells in control animals. No significant changes in water group. Significant changes in O_3_ in the water group.
Megele R. et al. (2018)	Case series	Glioblastoma: Primary (1) Recurrent (4)	Surgical re-resection + CT + intrathecal O_3_	Patients	5. 2 groups: - 1 primary - 4 recurrent	Increased survival rates compared to historical series not treated with O_3_. The patient treated immediately after the first surgery is alive and without recurrence.
Clavo B. et al. (2004)	Case-control series	Advanced head and neck tumors	RT + 5-FU RT + O_3_ (rectal insufflation, autohemotherapy)	Patients	19. 2 groups: - 12 (RT + 5-FU) - 7 (RT + O_3_)	Same survival rates in both groups.
Borreli E. et al. (2012)	RCT single-blind	Lung cancer	CT CT + Viscum Alba (VA) + O_3_ (auto- hemotherapy)	Patients	40. 2 groups: - 20 CT - 20 CT + O_3_ + VA	O_3_ and VA therapy was safe and seems to improve the quality of Life (QLQ30) in advanced lung cancer patients when used in association with CT.

## Data Availability

Data is contained within the article.
